# Phenylpropanoid Metabolism in Astringent and Nonastringent Persimmon (*Diospyros kaki*) Cultivars Determines Sensitivity to *Alternaria* Infection

**DOI:** 10.1021/acs.jafc.1c01312

**Published:** 2021-05-13

**Authors:** Akhilesh Yadav, Anton Fennec, Rachel Davidovich-Rikanati, Sagit Meir, Bettina Kochanek, Efraim Lewinsohn, Asaph Aharoni, Noam Alkan, Haya Friedman

**Affiliations:** † Department of Postharvest Science of Fresh Produce, Agricultural Research Organization (ARO), Volcani Center, Rishon LeZion 7505101, Israel; ‡ Newe Ya’ar Research Center, Agricultural Research Organization (ARO), Ramat Yishay 3009500, Israel; § Department of Plant and Environmental Sciences, Weizmann Institute, Rehovot 7610001, Israel

**Keywords:** black spot disease, cultivar collection, persimmon extracts, polyphenols, procyanidin

## Abstract

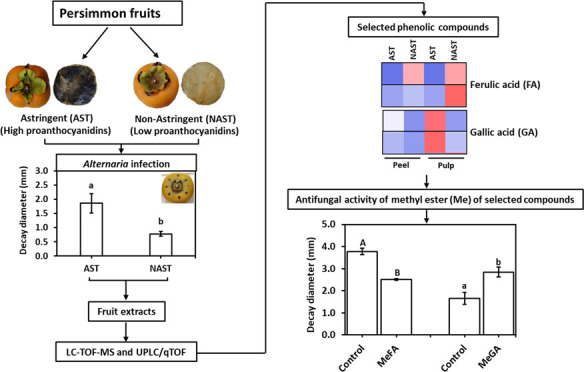

Fruits of nonastringent persimmon cultivars, as compared to astringent ones, were more resistant to *Alternaria* infection despite having lower polyphenol content. Metabolic analysis from the pulp of nonastringent “Shinshu”, as compared to the astringent “Triumph”, revealed a higher concentration of salicylic, coumaric, quinic, 5-*o*-feruloyl quinic, ferulic acids, β-glucogallin, gallocatechin, catechin, and procyanidins. Selected compounds like salicylic, ferulic, and ρ-coumaric acids inhibited *in vitro Alternaria* growth, and higher activity was demonstrated for methyl ferulic and methyl ρ-coumaric acids. These compounds also reduced *in vivo Alternaria* growth and the black spot disease in stored fruits. On the other hand, methyl gallic acid was a predominant compound in the “Triumph” pulp, as compared to the “Shinshu” pulp, and it augmented *Alternaria* growth *in vitro* and *in vivo*. Our results might explain the high sensitivity of the cultivar “Triumph” to *Alternaria*. It also emphasizes that specific phenolic compounds, and not the total phenol, affect susceptibility to fungal infection.

## Introduction

The persimmon cultivar (*Diospyros kaki* L. var. “Triumph”) is a major commercial cultivar in Israel, but it is sensitive to a necrotrophic fungus *Alternaria* that causes the black spot disease in many plant species. Various commercially available fungicides^1^ can inhibit *A. alternata* infection; however, the trend of zero tolerance to chemical fungicides, particularly during postharvest, makes the struggle against *Alternaria* very challenging.^2^ This trend greatly impacts stored fruits such as the persimmon cultivar “Triumph”, which is stored for up to 3 months and suffers major losses due to *Alternaria* development in storage.^3^

Metabolites produced by resistant plants including phenols can fulfill the role of a natural antifungal compound since many of the natural polyphenols are considered as Generally Recognized As Safe (GRAS).^4^ Plant phenols include several major groups: phenylpropanoids, flavonoids, phenolic acids, tannins (hydrolyzable (HT) and condensed (CT)), stilbenes, and lignans.^5^ Most of the investigated phenols exhibited inhibition effects on fungi and other microorganisms.^5^ This effect can be attributed to enhanced plant resistance as seen for salicylic acid,^6^ but can also result from a direct effect on the fungus. Tannins^7^ and flavonoids^8^ are especially known for their antifungal activity, and the polyphenol structure can affect the antifungal activity.^9^

Plant tannins are responsible for astringency in fruits,^7^ and CTs are more astringent than HT.^10^ Astringency is sensed as oral puckering/dry perception in the mouth, and it is believed to be caused by the aggregation and binding of saliva proteins on the surface of the tongue.^7^ In commerce, astringency is removed by exposure to high CO_2_ levels.^11^ Proanthocyanidins are the precursors of CT, and they are produced via the flavonoid biosynthesis pathway initiated from phenylalanine.^12^

Most persimmon cultivars are astringent, but few are nonastringent. The genetic trait of astringent/nonastringent of the Japanese cultivars is controlled by a single recessive locus (AST/ast).^12^ It was established that the proanthocyanidins stop accumulating 6–7 weeks after bloom, leading to a nonastringent phenotype, concomitant with the expression arrest of genes of the flavonoid pathway,^13^ and repression of *MYB4*.^14^

Although the origin of persimmon is from the Far East, many cultivars are growing in the Mediterranean region.^15^ During the 1990s, nonastringent (Japanese origin) and astringent (Chinese origin) cultivars were introduced to Israel.^16,17^ These cultivars constitute the persimmon collection at the Agriculture Research Organization-Volcani Center, Israel, and so far, it includes 17 persimmon cultivars.^18^ In this study, the susceptibility to *A. alternata* of 16 cultivars was examined. In addition, the phenol, soluble tannin content, and antifungal activity of extracts from these cultivars were determined. Furthermore, the polyphenol metabolites in representative astringent Cv. “Triumph” and nonastringent Cv. “Shinshu” were assessed. The antifungal activity of pure selected compounds, which exhibited differential levels between astringent and nonastringent persimmon cultivars against *Alternaria alternata*, were determined through *in vitro* and *in vivo* methods.

## Materials and Methods

### Plant Materials

Fruits of all persimmon cultivars were obtained from a 12 year old orchard planted at Agricultural Research Organization (ARO)-Volcani Center, Israel (31°59′25″ N, 34°49′2.84″ E). The list of cultivars is depicted in Table S1. The fruits were harvested during 2015–2017 between August and December. Green-orange fruits of a similar maturity stage for each of the cultivar were harvested, and their quality parameters have been described elsewhere.^18^ The fruits used for either *Alternaria* tests or phenolic extractions were disinfected with Taharsept solution (500 ppm, latent available chlorine-LAC, Israel) and set to dry.

### Evaluation of Black Spot Disease after Storage

The fruits of persimmon cultivars “Triumph” with a minor infection under the sepals were used to examine the effect of chemicals on black spot disease developed during storage and caused by *Alternaria*. Those fruits were dipped in solutions of different compounds/chemicals for 30 s. The chemical reagents were salicylic acid (SA), ferulic acid (FA), ρ-coumaric acid (ρCA), methyl ρ-coumaric acid (MeCA), and methyl ferulic acid (MeFA), (Sigma Aldrich, Israel; TCI Co., Japan). All chemicals were dissolved in absolute methanol, and 1 mM working concentration (containing 2% (v/v) methanol) was prepared in distilled water (DW). This concentration was chosen following preliminary experiments to choose the lowest effective concentration. Following dipping, the fruits were stored at 0 °C for 3 months. Control fruits were treated with 2% (v/v) methanol. Black spot disease severity was scored by a scale with a category from 1 to 5 (Figure S1), independently for the top and the bottom of the fruit as previously described.^19^

### *A. alternata* Fruit Inoculation

*A. alternata* conidia were cultivated on potato dextrose agar (PDA) containing 50 mg/mL chloramphenicol. The cultures were incubated at 22 °C for 7–14 days in the darkness. Conidia were collected from mature cultures, and the final working concentration was adjusted to 10^5^ conidia/mL. Sterilized fruits of Cv. “Triumph” were pierced in eight spaced-apart spots, creating a 1 mm hole. Each wound was inoculated with 7 μL of conidia suspension. Decay diameter (mm) and infection incidence (%) were measured following 5 days at 24 °C and 90–95% relative humidity.

To examine the efficacy of the pure chemicals, 7 μL of 1 mM of the compounds SA, ρCA, FA, MeCA, MeFA, and methyl gallic acid (MeGA) was added to each hole, followed by the addition of 7 μL of conidia suspension. In addition, the efficacy of MeGA was examined by preincubation of 1 mM MeGA with conidia suspension for 4 h. In an additional test, the fruits were first dipped in 1 mM MeGA for 2 min, then pierced, and inoculated with *A. alternata*.

### *In Vitro* and *In Vivo* Antifungal Activities of Pure Compounds

The antifungal activity of the pure compounds was examined by following the fungal growth in potato dextrose broth (PDB) and by monitoring conidial germination.^20^ Conidia suspension of *A. alternata* (10^5^ conidia/mL) in PDB media (200 μL) was loaded into a 96-well plate. The compounds SA, FA, ρCA, MeCA, and MeFA, in addition to (−)-epigallocatechin (epiCAT), (−)-gallocatechin (galCAT), gallic acid (GA), and MeGA, were added to the PDB media at 1 mM. Plates were incubated at 22 °C with rotation, and the absorbance at 600 nm was monitored for 64 h by a microplate reader (EnSpire 2300, Perkin Elmer). The experiments were performed at least three times with eight replicates for each treatment with similar results, and one experiment was presented.

The germination assay was performed by adding the pure selected chemicals at a final concentration of 1 mM to a freshly prepared conidia suspension in a PDB medium. Following 16 h at 22 °C, conidia were washed with phosphate buffer (PBS) and stained with SYTOX green dye (Thermo Fisher Scientific) at a final concentration of 50 nM in PBS. Conidia were observed with a fluorescent microscope (Olympus BX53, Japan) under 460/500 nm excitation and a 510 nm GFP emission filter. The number of germinated/nongerminated, dead, or alive conidia was scored, and the percentage was determined for each observation. A total of 50 conidia were scored for each observation, and at least three independent examinations were performed for each treatment.

### Extract Preparations and Determination of Total Phenols and Condensed Tannins

Extracts were prepared either from fruit wedges containing peel and pulp or from only pulp or peel of persimmon cultivars. Fresh tissue (4–5 g) was homogenized (Kinematic CH6010, Switzerland) in 20 mL of extraction solvent (70% methanol (v/v), 30% acetone (v/v), and 0.03% butylated hydroxytoluene (w/v)). Extracts were incubated for 60 min on an orbital shaker at room temperature (RT) and centrifuged at 15 000*g* (Sorvall RC6, Thermo Fisher, Germany) at 4 °C for 15 min. The supernatant was collected, and the pellet was resuspended with 10 mL of the extraction solvent. The supernatants were combined and dispensed into 2 mL Eppendorf tubes and stored at −80 °C for further use. Total phenol, proanthocyanidins, and anti-*Alternaria* activity were measured in extracts prepared from fruit wedges. Anti-*Alternaria* activity was also measured in extracts prepared from only the peel.

Total phenol concentration was determined using a modified Folin–Ciocalteu method.^21^ The reaction was carried out by mixing 100 μL of the phenolic extract with 7.9 mL of DW and 500 μL of the Folin–Ciocalteu reagent. The reaction mixture was incubated for 1 min; then, sodium carbonate (20%; 1.5 mL) was added and incubated in the darkness for 2 h at RT. Absorbance was measured at 765 nm using a spectrophotometer (Ultrospec 2100 pro, Biochrom). The calibration curve was based on gallic acid (GA) dissolved in a fresh extraction solvent at increasing concentrations (0, 0.25, 0.5, 1, 1.5, 2, 2.5, 5, 10, 15 mg/mL), *R*^2^ = 0.96.

Proanthocyanidins (condensed tannins), concentration was measured using the acidic vanillin reaction method.^22^ The sample extract of 50 μL (diluted 1:1 in the extraction solvent) was mixed with 3 mL of 4% (w/v) vanillin dissolved in methanol. HCl (37%; 1.5 mL) was added, and the reaction was incubated for 15 min. The absorption was measured at 500 nm. The calibration curve was based on catechin (CAT) dissolved in a fresh extraction solvent at increasing concentrations (0, 0.25, 0.5, 1, 1.5, 2, 2.5, 5, 10, 15 mg/mL), *R*^2^ = 0.99. Measurements of total phenol and proanthocyanidin concentrations were performed on three biological samples each of three technical repetitions.

### Determination of Extract Bioactivity against *A. alternata*

Two bioactivity assays were used to assess the *A. alternata* growth inhibition as schematically represented in Figure S4. The paper disc method^23^ was performed on PDA Petri plates, which were inoculated with 300 μL of conidia suspension. Sterile Whatman paper discs were placed on each plate; the control contained 100 μL of fresh extraction solvent, while others contained 100 μL of extracts from different cultivars. Before loading the extract on the Whatman discs, each extract was adjusted according to the fresh weight used for the extraction. The plates were incubated for 5 days at RT, and the inhibition/halo zones were measured.

The fungal growth inhibition was evaluated also by the “poisoned media” assay.^24^ PDA Petri plates (60 mm) containing 1 mL of extract/plates and a fresh extraction solvent were used as a control. *A. alternata* plugs (5 mm diameter) were placed in the center of the plate, the diameter of *A. alternata* growth was measured following 5 days of incubation at RT, and the net growth in comparison to the control was calculated. All bioactivity tests were performed on three biological samples each of three technical repetitions.

### Metabolite Analysis

The determination of the metabolites was performed on the fruits of persimmon cultivars “Triumph” and “Shinshu”. Samples of peel or pulp were extracted as described above and were used for the metabolomics analysis by two independent platforms. In the first platform, samples were subjected to liquid chromatography/time-of-flight/mass spectrometry (LC-TOF-MS). Methanolic extracts were filtered through Acrodisc syringe filters with a GHP membrane, 13 mm with 0.2 μm (PALL), and transferred to vials for liquid chromatography/time-of-flight/mass spectrometry (LC-TOF-MS) analysis, which was carried out on Agilent 1290 Infinity series liquid chromatography coupled with an Agilent 1290 Infinity DAD and an Agilent 6224 Accurate Mass Time of Flight (TOF) mass spectrometer (MS) (Agilent Technologies, Santa Clara). Compounds were separated on a Zorbax Extend-C18 Rapid Resolution HT column (2.1 × 50 mm^2^, 1.8 μm; Agilent Technologies). The gradient elution mobile phase consisted of H_2_O with 0.1% (v/v) formic acid (eluent A) and acetonitrile containing 0.1% (v/v) formic acid (eluent B). The column was equilibrated with 2.5% eluent B at a flow rate of 0.3 mL/min for 1.5 min. Eluent B was then increased to 80% until 11 min and maintained until 13 min and then raised to 95% B until 14 min and restored to 5% by 17 min for re-equilibration until 19 min. The flow rate of the mobile phase was 0.3 mL/min, and the column oven temperature was 40 °C. Eluted compounds were subjected to the Jet Stream electrospray ionization interface operated in the negative mode with the following settings: gas temperature of 300 °C with a flow of 11 L/min and a nebulizer set to 30 psig and a sheath gas temperature of 300 °C at 12 L/min flow. VCap was set to 3000 V, the fragmentor to 140 V, and the skimmer to 65 V. Phenolic compounds found in persimmon were targeted and integrated according to published data.^25^ The main (therefore representative) ions formed in the ESI source (mainly [M – H], [M + Cl]^−^, and [M + HCOO]^−^) of target compounds were detected using the “find compound by the formula” function and analyzed by Mass Hunter qualitative and quantitative analysis software version B.07.00 (Agilent technologies). Compounds’ identities were annotated by comparison of exact molecular mass (EMM) to their theoretical mass. When standards were available, the retention time (RT) of purchased authentic standards (AS) was also compared.

In the second platform, the fruit extracts were dried to 30% and an aliquot of 100 μL was mixed with 50 μL of double DW, 100 μL of hexane, and a final concentration of 0.1% formic acid. The samples were vortexed for 1 min, placed on an ice vortex again, and then centrifuged at 13 000*g* for 10 min at 4 °C. The lower phase was taken and filtered before characterization. Samples were analyzed as described^26^ using a high-resolution UPLC/qTOF system comprising a UPLC (Waters Acquity) connected to a qTOF detector (tandem quadrupole/time-of-flight mass spectrometer, Waters). Separation of metabolites was performed on a 100 × 2.1 mm^2^, 1.7 μm UPLC BEH C18 column (Waters Acquity). The mobile phase consisted of 0.1% formic acid in acetonitrile/water (5:95, v/v; phase A) and 0.1% formic acid in acetonitrile (phase B). Metabolites were identified by comparing the retention times and mass fragments of standard compounds. When the corresponding standards were not available, compounds were putatively identified by comparing their retention times, elemental composition, and fragmentation pattern with those described in the literature.^27,28^

All annotated compounds from both platforms are listed in Tables S2 and S3 for the first platform and Tables S4 and S5 for the second platform. The integrated peak area (PA) of the main ion produced was used for the quantitative analyses. Results are expressed as peak area and as a percentage of each one out of the total. The data are presented as a heat map using the Heatmapper online tool (http://www.heatmapper.ca/).

### Statistics Analysis

Statistical analyses were performed with SAS JMP Pro 13.0 (2016). Data were analyzed by one-way ANOVA, and the mean differences were tested by the Tukey–Kramer HSD test at *p* ≤ 0.05, followed by a normal distribution test. Different letters indicate a significant difference between treatments. Differences in antifungal activities among the compounds expressed as dead/alive and germinated/nongerminated conidia were statistically evaluated with Pearson and likelihood ratio chi-square multiple comparison tests.

## Results

### Astringent Persimmon Cultivars are More Sensitive to *Alternaria* than Nonastringent Cultivars

**Figure 1:**
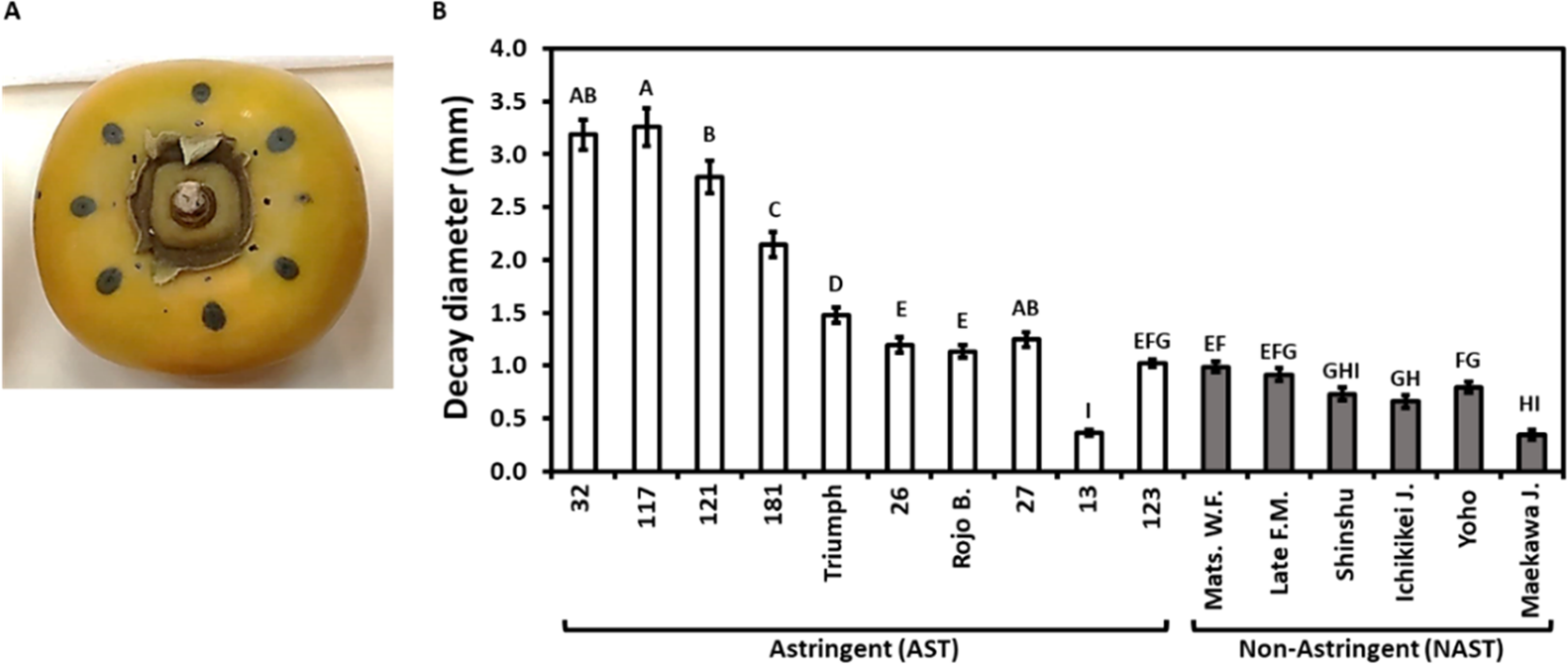
Fruit decay by *A. alternata* infection on the different persimmon cultivars. Mature fruits of different cultivars were pierced and inoculated with *A. alternata* (10^5^ conidia/mL) (A); decay diameter was measured on 2016 fruits following 5 days of incubation at RT (B). The bars represent the average of 8 sites on 14 independent fruits for each cultivar ± SE. Different letters indicate a significant difference between treatments according to Tukey–Kramer HSD; *p* ≤ 0.05. Rojo B.; Rojo Brillante, Mats. W.F.; Matsumoto Wasa Fuyu, Late F.M.; Late Fuyu Mutant, Ichikikei J.; Ichikikei Jiro, and Maekawa J.; Maekawa Jiro.

Ten astringent Chinese cultivars and six nonastringent Japanese cultivars from the ARO^18^ collection were used in this study. The sensitivity to *A. alternata* of all cultivars was examined *in vivo* in the years 2015 (data not shown) and 2016, by placing a drop containing *A. alternata* conidia into a wound on the upper surface of the fruit (Figure 1A,B). The decay diameter developed by the *A. alternata* was similar for both years. Five out of 10 astringent cultivars (Cv. “32”, “117”, “121”, “181”, and “Triumph”) exhibited a higher decay diameter than that of the nonastringent cultivars. The average decay diameter on the astringent cultivars was 1.86 ± 0.34 and that of the nonastringent one was 0.78 ± 0.089 (Student’s *t*-test; *p* ≤ 0.05).

### Anti-*Alternaria* Activity, Polyphenol, and Soluble Tannins of Persimmon Extracts

**Figure 2:**
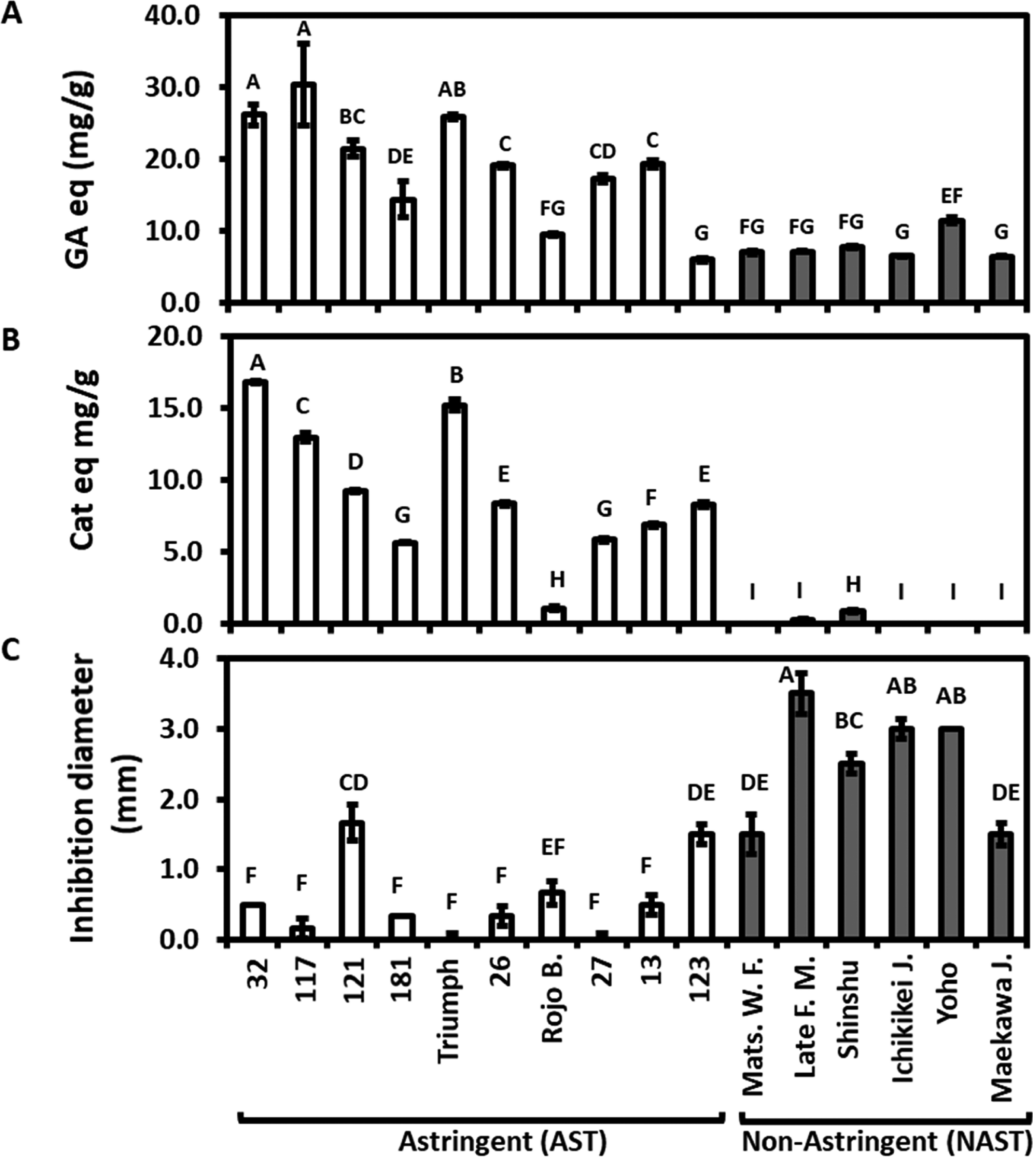
Total phenols, proanthocyanidin, and anti-*Alternaria* activity in the astringent and nonastringent persimmon cultivar extracts. Extracts were prepared from fruit wedges, and total phenol (A) was determined as gallic acid (GA) equivalents, proanthocyanidin (B) as CAT equivalents, and the inhibition diameter (C) was measured following 5 days at RT by the disc assay. Control discs did not inhibit *A. alternata*. The bars are an average of three independent extractions each performed on four replicates ± SE. Different letters indicate a significant difference between treatments according to Tukey–Kramer HSD; *p* ≤ 0.05. Rojo B.; Rojo Brillante, Mats. W.F.; Matsumoto Wasa Fuyu, Late F.M.; Late Fuyu Mutant, Ichikikei J.; Ichikikei Jiro, and Maekawa J.; Maekawa Jiro.

Extracts were prepared from wedges of the 16 persimmon cultivars. The levels of total phenols (GA-equivalents) and proanthocyanidin (CAT equivalents) were determined (Figure 2A,B). The astringent cultivars contain, by far, higher levels of total phenols and proanthocyanidin, except for the extract of Cv. “Rojo Brillante”, which contained low levels of both.

The anti-*Alternaria* activity of the extracts was evaluated *in vitro* by the paper disc assay. The inhibition diameter of *A. alternata* growth caused by paper discs soaked with persimmon extracts was larger for the nonastringent cultivars than for the astringent cultivars (Figure 2C). In this assay, the extracts of most of the astringent cultivars (8 out of 10) had a lower anti-*Alternaria* activity than the majority of the nonastringent cultivars. The average inhibition/halo diameter of the nonastringent cultivars was five times higher than that of the astringent ones (2.55 ± 0.33 mm diameter of nonastringent in comparison to 0.59 ± 0.18 mm of astringent; Student’s *t*-test; *p* ≤ 0.05). Hence, extracts of nonastringent cultivars had significantly higher anti-*Alternaria* activity (Figure 2C). This anti-*Alternaria* activity was inversely related to the polyphenol/proanthocyanidin levels of the extracts (Figure 2A,B), suggesting that there are specific antifungal compounds in nonastringent cultivars or fungal-promoting compounds in astringent cultivars.

**Figure 3:**
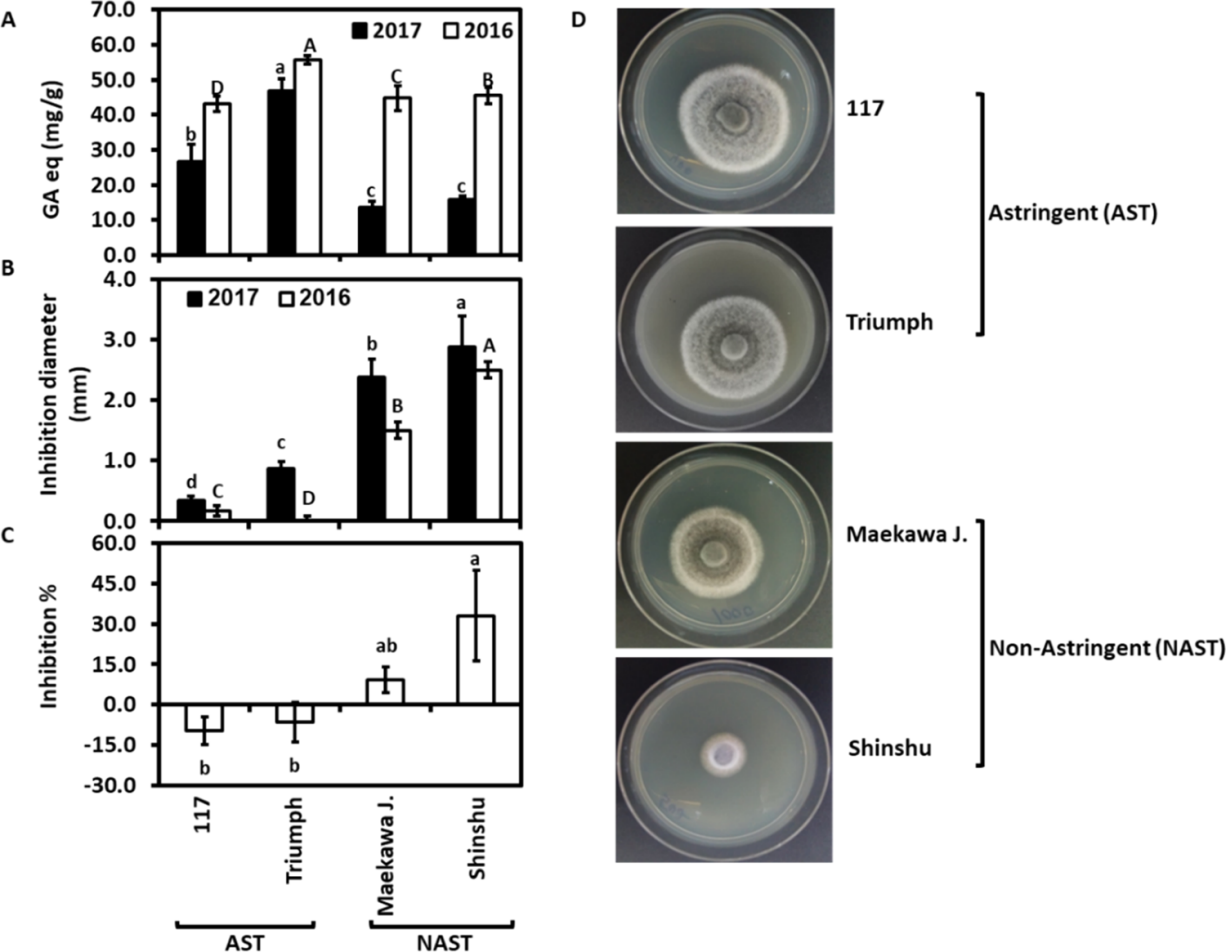
Total phenol and anti-*Alternaria* activity in extracts of Cvs. “Triumph” and “117” (astringent) and “Maekawa Jiro” and “Shinshu” (nonastringent). Total phenol (A) and anti-*Alternaria* (B–D) activities were measured in extracts from peel by the disc assay (B) and by the poisonous media assay (C, D), expressed as percent inhibition in comparison to the control. The results were recorded following 5 days in both assays. The results are an average of 3 extracts ± SE. Different letters indicate a significant difference between treatments according to Tukey–Kramer HSD; *p* ≤ 0.05. Rojo B.; Rojo Brillante, Mats. W.F.; Matsumoto Wasa Fuyu, Late F. M.; Late Fuyu Mutant, Ichikikei J.; Ichikikei Jiro and Maekawa J.; Maekawa Jiro.

Extracts were prepared from the peel of two astringent cultivars “Triumph” and “117” and two nonastringent cultivars “Shinshu” and “Maekawa Jiro”. Total phenol determination in extracts at both years confirmed that the astringent cultivars had higher phenol content than the nonastringent cultivars; in 2016, the levels were about 30% higher, while in 2017, it was about 400% higher (Figure 3A). The anti-*Alternaria* activity of these extracts was examined *in vitro*, using the paper disc assay (Figure 3B) and the poisonous media assay (Figure 3C,D). Both nonastringent Cvs. “Maekawa Jiro” and “Shinshu” had higher anti-*Alternaria* activity than astringent Cvs. “Triumph” and “117”, while Cv. “Shinshu” had the highest activity in both years. Note that in the year 2017, Cvs. “Shinshu” and “Maekawa Jiro” contained low levels of phenols compared to that in 2016, but the anti-*Alternaria* activity was the highest. The poisonous media assay enabled us to determine that both the astringent cultivars “Triumph” and “117” contained compounds that enhanced the *A. alternata* growth (Figure 3C).

### Identification of Specific Compounds in Astringent and Nonastringent Persimmon Cultivars

**Figure 4:**
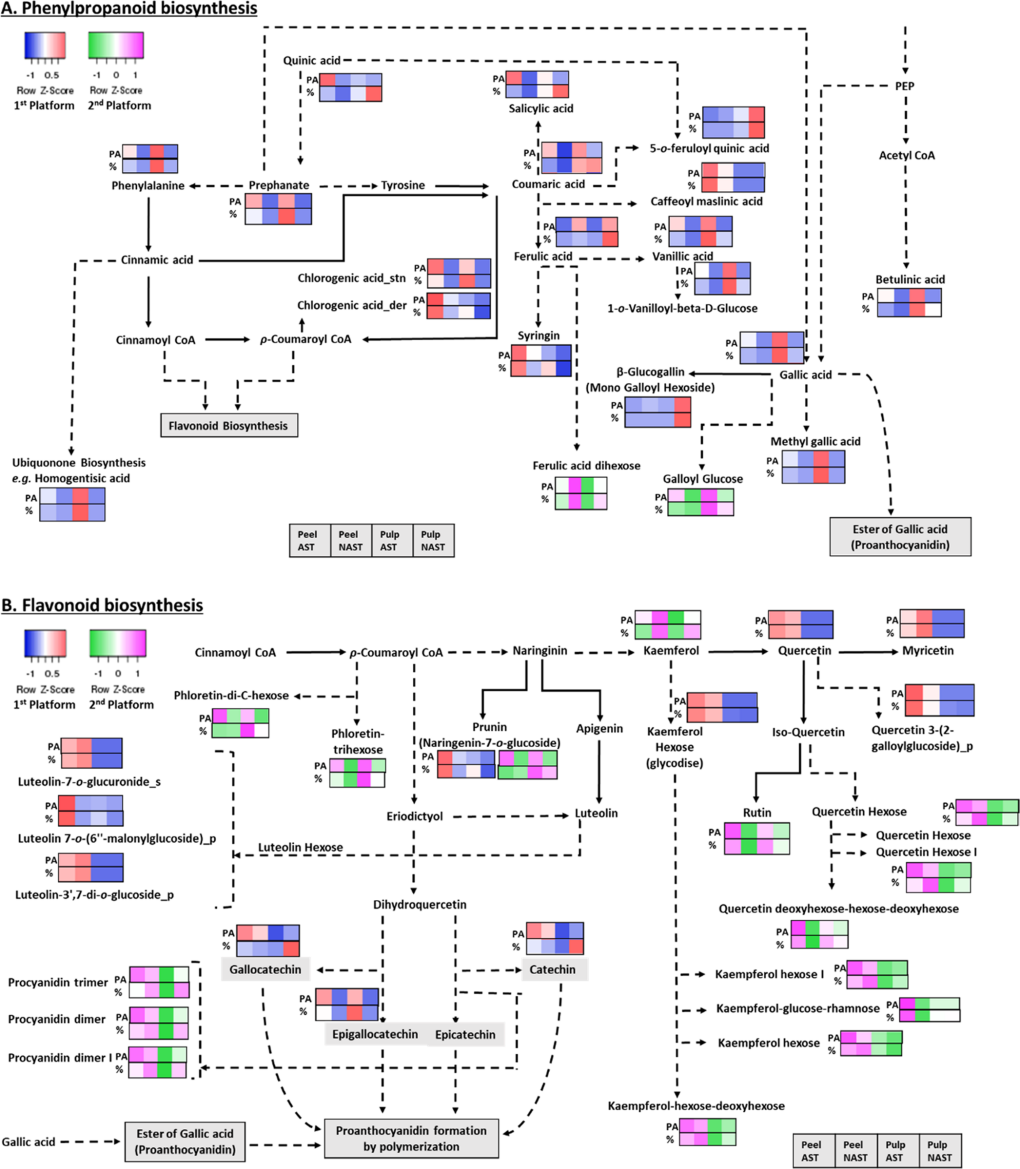
Polyphenols in peel and pulp extracts of Cvs. “Shinshu” (nonastringent/NAST) and “Triumph” (astringent/AST). Polyphenols were determined by two platforms, and relative levels for each compound were determined by the area under the peak of each compound (PA) and by calculating the percentage of each compound out of total compounds (%). Phenylpropanoid pathway (A); flavonoid and flavone pathways (B). Comparisons of blue and red are based on data from the first platform, while those of green and pink are based on data from the second platform. Heatmapper online tool (http://www.heatmapper.ca/) was used to create these comparisons. The position of each square in these comparisons from left to right shows peel AST, peel NAST, pulp AST, and pulp NAST. Solid lines indicate a direct interaction and the dashed lines indirect.

To uncover the compounds that contribute to the antifungal activity in Cv. “Shinshu”, the composition of polyphenolic compounds (Figure 4) was determined in the peel and pulp of the astringent Cv. “Triumph” and the nonastringent Cv. “Shinshu”. The compounds and their peak areas, as determined by two separate platforms, are presented in Tables S2 and S3 for the first platform and in Tables S4 and S5 for the second platform. The comparisons between peel and pulp of these cultivars are presented as the peak area and the percent of each compound out of the total (Figure 4).

Fifty-one different compounds were identified in the peel and pulp of astringent/nonastringent persimmon by the two platforms, in which 45 were identified in the presented pathway scheme (Figure 4A,B). Each of the platforms identified different compounds, except for naringenin-7-*o*-glucoside, which was identified by both platforms. Eighteen compounds were identified within the phenylpropanoid pathway and eleven in the flavonoid and flavone biosynthesis pathway with the first platform (presented in blue/red) and two and fourteen, respectively, in the second platform (presented in green/pink) (Figure 4). Six compounds (11.76% of total) in the phenylpropanoid pathway and 19 (37.25% of total) in the flavonoid pathway were the highest in the peel of astringent Cv. “Triumph” in comparison to the other samples. The levels of only two compounds were similar by the first platform in both peel and pulp of astringent Cv. “Triumph”, i.e., prephenate and epigallocatechin. The levels of the compounds phenylalanine, coumaric acid, vanillic acid, 1-*o*-vanillioyl-β-d-glucose, gallic acid, methyl gallic acid, galloyl glucose, homogentisic acid, betulinic acid, and phloretin trihexose were the highest in the pulp of astringent Cv. “Triumph”. Hence, in total, 35 (68.62% of total) compounds were higher in astringent Cv. “Triumph” in comparison to nonastringent Cv. “Shinshu”, and most of them were the highest in the peel. Nevertheless, among the 25 compounds that were the highest in the peel of astringent Cv. “Triumph”, 12 appeared also in the nonastringent Cv. “Shinshu” and all were from the flavonoid pathway.

Several compounds appeared to be higher in the peel of nonastringent Cv. “Shinshu” in comparison to astringent Cv. “Triumph”, i.e., ferulic acid dihexose, myricetin, luteolin-3′,7-di-*o*-glucoside, and luteolin-7-*o*-glucuronide. The compound ferulic acid had similar levels in both the peel and pulp of nonastringent Cv. “Shinshu” and higher than that in the astringent Cv. “Triumph”. Based on the determination of percentage in both platforms, the compounds salicylic acid, ferulic acid, coumaric acid, quinic acid, 5-*o*-feruloyl quinic acid, and β-glucogallin of the phenylpropanoid pathway and gallocatechin and catechin of the flavonoid pathway had higher levels in the pulp extract of nonastringent Cv. “Shinshu” than in the other extracts. Procyanidin dimers or trimers had similar relative levels in the peel and pulp of nonastringent cultivars and higher than in astringent cultivar tissues. By and large, the compounds showing relatively high levels in nonastringent Cv. “Shinshu” are of the phenylpropanoid pathway and they are the highest in the pulp. In contrast, gallic acid and epigallocatechin, which are the precursors for proanthocyanidin, were much higher in astringent Cv. “Triumph” than in nonastringent Cv. “Shinshu”.

### Antifungal Activity of Specific Compounds Identified in Nonastringent Cultivar

**Figure 5:**
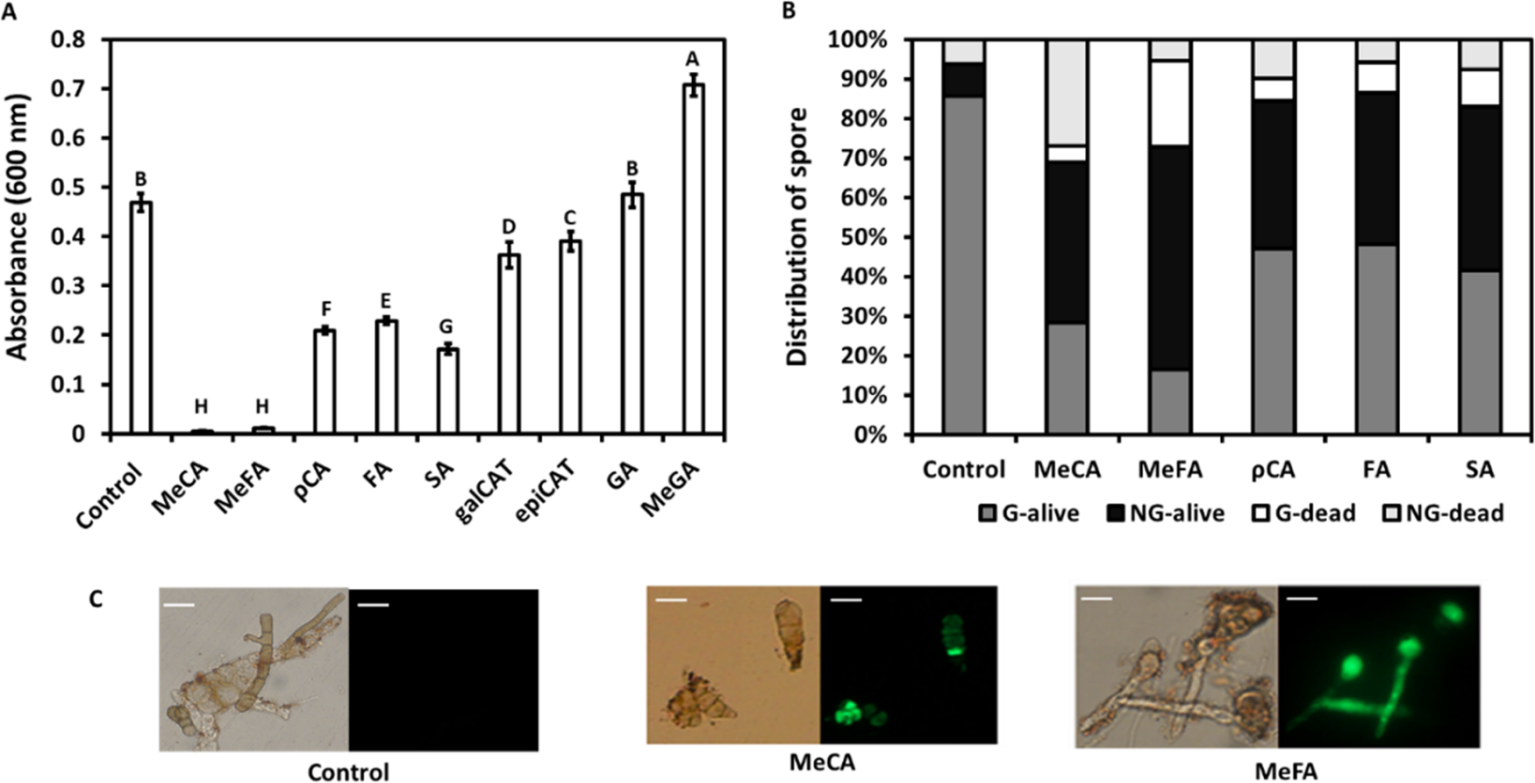
Effect of phenolic compounds on hyphae growth and conidia germination of *A. alternata*. The growth assay (A) was performed in liquid PDB media. Conidia and compounds (1 mM) were incubated at 22 °C for 64 h, and the absorbance at 600 nm was recorded. Results represent one experiment out of three experiments with an average of 8 replicates ± SE. Dead cells (B, C) were detected by SYTOX green dye. The dead or alive germinated (G)/nongerminated (NG) conidia were observed under 600× magnification following incubation for 16 h at 22 °C; scale bars are 100 μm. Representative pictures showing the effect of MeCA and MeFA in comparison to the control (C). Bright-field images (left) and fluorescence images (right) showing SYTOX green uptake of dead cells. Different letters indicate a significant difference between treatments according to Tukey–Kramer HSD; *p* ≤ 0.05 (A), and Pearson and likelihood ratio chi-square multiple comparison tests were applied for (B) showing a statistical difference at *p* ≤ 0.05.

Few of the compounds exhibiting relatively higher levels in nonastringent Cv. “Shinshu” were obtained as pure compounds, and their activity was measured by determining the growth curve of *A. alternata* in a liquid medium (Figure 5A). The compounds FA, ρCA, and SA had a moderate effect on *A. alternata* growth and reduced the growth by about 50%. However, the methylated forms of FA and ρCA completely inhibited *A. alternata* growth. The (−)-epigallocatechin and catechin, which had a relatively higher percentage in the fruits of nonastringent cultivars, had only minor antifungal activities. On the other hand, the compounds GA and MeGA, which exhibited higher levels in the astringent Cv. “Triumph”, did not inhibit the growth, and the MeGA, even, augmented growth.

To examine the effect of these compounds on germination, *A. alternata* conidia were incubated in the presence of the selected compounds. In the control, about 85% of the conidia were germinated and alive, while the rest did not germinate and half were dead (Figure 5B,C). ρCA, FA, and SA reduced conidia germination by more than a half, consequently increased the nongerminated conidia, and about 85% of the nongerminated conidia were alive. MeCA and MeFA further reduced the conidia germination (only 15–25% germinated) and increased the percentage of dead cells. While MeCA increased the percentage of dead nongerminated conidia, MeFA increased the percentage of dead germinated conidia, suggesting a slightly different mechanism of action of these compounds.

The antifungal activity assay was performed also *in vivo* (Figure S2). The fruits of the cultivar “Triumph” were pierced and inoculated with a conidial suspension of *A. alternata* and with different compounds. All compounds reduced the decay diameter of *Alternaria* in comparison to the control. The methyl forms of ρCA and FA were the most effective in inhibiting *Alternaria* growth and decreased the infection incidence. The MeGA did not enhance the growth using this procedure, neither in Cvs. “Triumph” nor in “Shinshu”. However, the MeGA enhanced the *A. alternata* growth when fruits of Cvs. “Triumph” and “Shinshu” were inoculated with *A. alternata*, which was preincubated with MeGA, or the fruit was first dipped in MeGA and then inoculated (Figure S3A,B). Incubation for 2 min of Cv. “Shinshu” with MeGA (1 mM) also enhanced the fruit-softening following 5 days at ambient temperature (14% soft fruit of control and 42% for the MeGA-treated fruit; data not shown).

**Figure 6:**
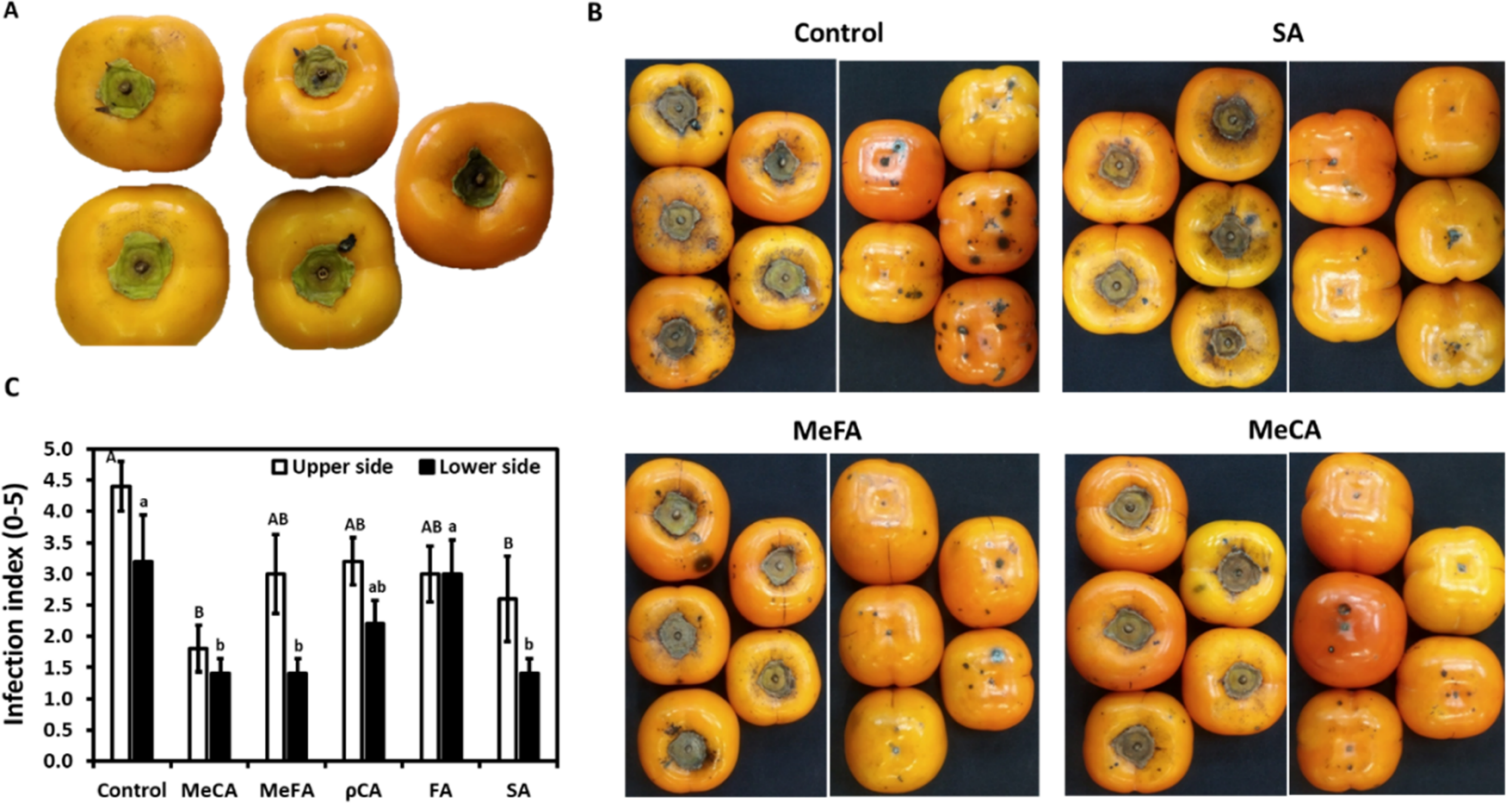
Effect of selected polyphenol compounds on black spot disease. The fruits of cultivars “Triumph” with quiescence infection (A) at harvest were dipped with MeCA, MeFA, ρCA, FA, and SA at 1 mM for 2 min. Following 3 months of storage at 0 °C (B), fruits were monitored for black spot disease (C), according to the severity scale presented in Figure S1. Results present an average of 5 fruits ± SE. Different letters indicate a significant difference between treatments according to Tukey–Kramer HSD; *p* ≤ 0.05. Representative fruits (B) from the different treatments are presented.

In addition, the effect of the compounds was evaluated also on the development of black spot disease during storage. The fruits of astringent Cv. “Triumph” exhibiting a low level of quiescence infection at harvest on the top of the fruits were dipped in different compounds’ solution and stored at 0 °C for 3 months (Figure 6A,B). Black spot disease was evaluated at the top and the bottom of the fruits by categorizing the disease according to a scale of severity (Figures 6C and S1). All of the chemicals reduced the infection index at the top and bottom of the fruit, except for FA, which was less effective at the bottom. The MeCA was the most effective in reducing the black spot disease both at the top and at the bottom of the fruit.

## Discussion

*A. alternata* is a common pathogen of persimmon cultivation and postharvest handling.^29^ Physical parameters like cuticle or cell wall,^30^ as well as naturally synthesized or induced endogenous chemicals,^31^ can affect the sensitivity of a crop to a pathogen. In this study, there was genetic variability in the anti-*Alternaria* natural compounds among the persimmon cultivars. This was based on the assay using the pierced peel of freshly harvested fruit, showing different infection severity with *Alternaria*. Therefore, this study concentrated on phenolic compounds that directly enhance or reduce *Alternaria* growth. Persimmon fruits are rich in polyphenol,^32^ and two platforms were used to identify these compounds. This study showed that the two platforms yielded a different set of compounds (Figure 4). A large-scale metabolic analysis was performed in melon using seven different platforms, and each contributed to the overall array of metabolites.^33^ Different methods of sample handling and identification can yield different compounds.^32^ Hence, this study is in line with previous metabolic analyses and further emphasizes the importance of using multiple platforms for polyphenol identification.

Astringency in persimmon results from soluble tannins,^11^ and indeed, the levels of soluble tannins in the freshly harvested astringent cultivars were higher than in the nonastringent cultivars. In accordance with higher levels of tannin, also the total polyphenols were five times higher in astringent cultivars than in nonastringent ones (Figure 2). Nevertheless, *Alternaria* infection was lower in the nonastringent cultivars than in the astringent cultivars (Figure 1). Additionally, the anti-*Alternaria* activity of the extracts from nonastringent cultivars “Maekawa Jiro” and “Shinshu” was higher than those from astringent cultivars “Triumph” and “117” (Figures 2 and 3). This can be explained by either the existence of *Alternaria* augmenting compounds in astringent cultivars, or by anti-*Alternaria* compounds in the nonastringent cultivars, or by both. The comparison between peel and pulp of astringent and nonastringent cultivars revealed differences in their phenolic compounds (Figure 4). The extracts of astringent persimmon Cv. “Triumph” contained compounds that stimulated fungal growth (Figure 3D). Further analysis of the compounds revealed that methyl gallic acid was the highest in Cv. “Triumph”, and this compound augmented *Alternaria* growth in Cvs. “Shinshu” and “Triumph”, as was demonstrated by *in vitro* (Figure 5) and *in vivo* assays (Figure S3). This might explain the sensitivity of the leading Israeli persimmon cultivar “Triumph” to *Alternaria* infection, which develops during extended storage.^29^ Interestingly, gallic acid was reported to inhibit the growth of several plant fungi^34,35^ and even human fungi.^36^ Increasing the hydrophobicity of gallic acid by methylation usually decreased the antifungal effectiveness of this compound,^35^ and in this study, the methylated gallic acid compound had even a higher *in vitro* fungal growth activity (Figure 5) and also enhanced *in vivo* fungal growth (Figure S3).

The relatively enriched compounds in the pulp of nonastringent Cv. “Shinshu” were of the phenylpropanoid pathway: quinic acid, salicylic acid, coumaric acid, ferulic acid, 5-*o*-feruloyl quinic acid, and β-glucogallin, and of the flavonoid pathway: gallocatechin, catechin, and a few procyanidins. The compounds salicylic acid, ferulic acid, and ρ-coumaric acid were the highest in the nonastringent cultivar, but not (−)-gallocatechin and (−)-epigallocatechin, and inhibited *Alternaria* growth *in vitro*, mainly by preventing germination (Figure 5). In contrast to the findings in this study, salicylic acid did not inhibit the *in vitro* growth of *Alternaria* even at higher concentrations,^37^ but it inhibited *Alternaria* growth on jujube (*Ziziphus jujuba* Mill.) fruit, possibly by inducing the fruit defense response against fungal pathogens.^37^ Differences between studies could stem from using different *A. alternata* isolates. Although *Alternaria* infection can be reduced by enhancing the fruit resistance by compounds like β-aminobutyric acid or SA, compounds such as coumaric and ferulic acids that directly affect the germination of *Alternaria* would be useful.^37−39^ In addition to this study, coumaric acid was identified also in the extract of jujube fruit peel and it inhibited *in vitro Alternaria* growth, as well as black spot rot caused by *A. alternata*.^39^ On the other hand, ferulic acid was found to have antifungal activity against fungi affecting fruit at postharvest, but *Alternaria* was not included in that study.^40^

The methylated forms of both coumaric and ferulic acids augmented the *in vitro* and *in vivo* anti-*Alternaria* activities of these compounds by inhibiting conidia germination. In addition, methyl coumaric acid and methyl ferulic acid enhanced the death of nongerminated conidia and germinated conidia, respectively (Figure 5). An increase in cell death of nongerminated conidia of *Alternaria* by ethyl coumaric acid has been demonstrated earlier and suggested to occur due to membrane disruption.^41^ Not all methylated polyphenol exhibited higher antifungal activity than their nonmethylated compounds; for example, while the methylated ρ-coumaric acid exhibited a higher antifungal activity,^39^ the methylated compound of cinnamic acid and ρ-hydroxybenzoic acid did not exhibit a higher activity than the parental form.^42^

Taken together, the paper showed that the mutation in the astringent persimmon cultivar causing nonastringency also reduced the total polyphenols and enhanced the antifungal activity against *Alternaria* pathogen. This study identified the compounds of the phenylpropanoid and flavonoid pathways in the fruits of astringent Cv. “Triumph” and nonastringent Cv. “Shinshu”. Compounds that are high in astringent cultivar “Triumph” like methyl gallic acid enhanced the pathogen growth, while the compounds salicylic acid, coumaric acid, and ferulic acid, which were high in the nonastringent Cv. “Shinshu”, reduced the growth. This study emphasizes that the genetic modification affecting astringency may alter the biosynthesis pathways of phenylpropanoid and flavonoid compounds and modify the susceptibility to pathogens. The results support the notion that the antifungal activity of polyphenol is dependent on specific molecules and not on the total amount of polyphenol.^43^ Due to public concerns about chemical fungicide’s toxicity to the environment and consumers, there are increasing restrictions during recent on the use of chemical fungicides, especially during postharvest.^1^ Therefore, the approach of using plant-derived materials as a natural antifungal treatment is becoming more popular as a valid alternative to chemical fungicides. This study may lead to future treatments to control *Alternaria* infection in harvested crops.

## Supporting Information Available

Index used to characterize black spot disease (Figure S1); effect of phenolic compounds on the colonization of *A. alternata* (Figure S2); effect of methyl gallic acid (MeGA) on the colonization of *A. alternata* (Figure S3); schematic presentation of the methods used for the anti-*Alternaria* activity (Figure S4); list of cultivars studied and harvest dates (Table S1); polyphenolic compounds and their peak area identified in the first platform (Table S2); metabolites putatively identified in peel and pulp of persimmon fruit using UHPLC-TOF/MS analysis from the first platform (Table S3); polyphenolic compounds and their peak area identified in the second platform (Table S4); and metabolites putatively identified in peel and pulp of persimmon fruit using UPLC/qTOF-MS from the second platform (Table S5) (PDF)
