# Supplementary material for: Phenylpropanoid Metabolism in Astringent and Nonastringent Persimmon (Diospyros kaki) Cultivars Determines Sensitivity to Alternaria Infection
Source: J Agric Food Chem. 2021 May 13;69(20):5628–37. doi: 10.1021/acs.jafc.1c01312 (PMC8278483; doi:10.1021/acs.jafc.1c01312)
Supplement: Supplementary file 1 — jf1c01312_si_001.pdf [file jf1c01312_si_001.pdf]

Supporting information

Phenylpropanoid metabolism in astringent and non-astringent persimmon (*Diospyros kaki*) cultivars determines sensitivity to *Alternaria* infection

Akhilesh Yadav*[1], Anton Fennec*[1], Rachel Davidovich-Rikanati[2], Sagit Meir[3], Bettina Kochanek[1], Efraim Lewinsohn[2], Asaph Aharoni[3], Noam Alkan[1], Haya Friedman**[1]

[1]Department of Postharvest Science of Fresh Produce, Agricultural Research Organization (ARO), Volcani Center, Rishon LeZion, Israel

[2]Newe Ya'ar Research Center, Agricultural Research Organization (ARO), Ramat Yishay, Israel

[3]Department of Plant and Environmental Sciences, Weizmann Institute, Rehovot, Israel

***equal contribution**

****Corresponding author**

Haya Friedman**

Tel: +972 506220624

Email: hayafr@volcani.agri.gov.il; hayafr@agri.gov.il

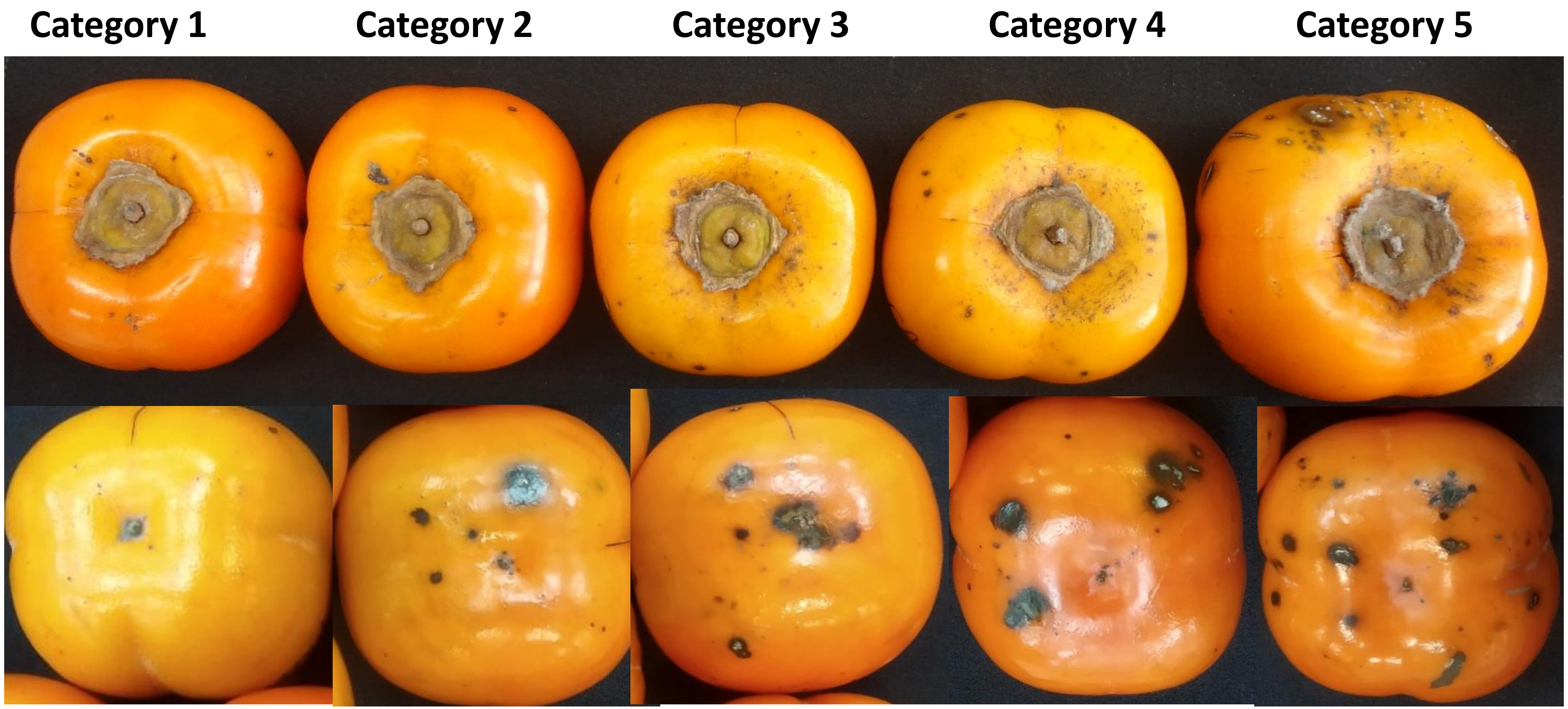

Figure S1. Index used to characterize black spot disease.

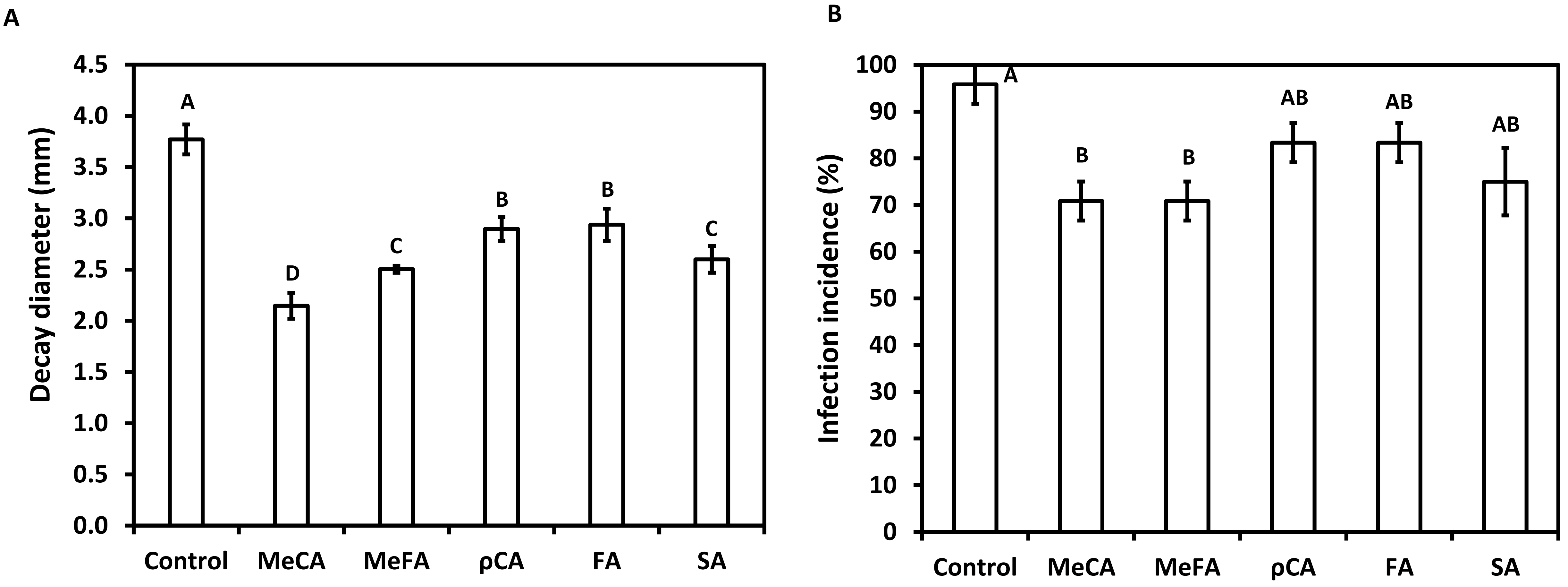

Figure S2. Effect of phenolic compounds on the colonization of *A. alternata*. The fruits of the cultivar ‘Triumph’ were pierced and inoculated with a conidia suspension of *A. alternata*, and the different compounds were added to the inoculation sites. Decay diameter (A) and the infection incidence (B) was evaluated. The values presented are the average of 3 fruit each with 8 infected sites ±SE. Different letters indicate a significant difference between treatments according to Tukey-Kramer HSD; $p \leq 0.05$.

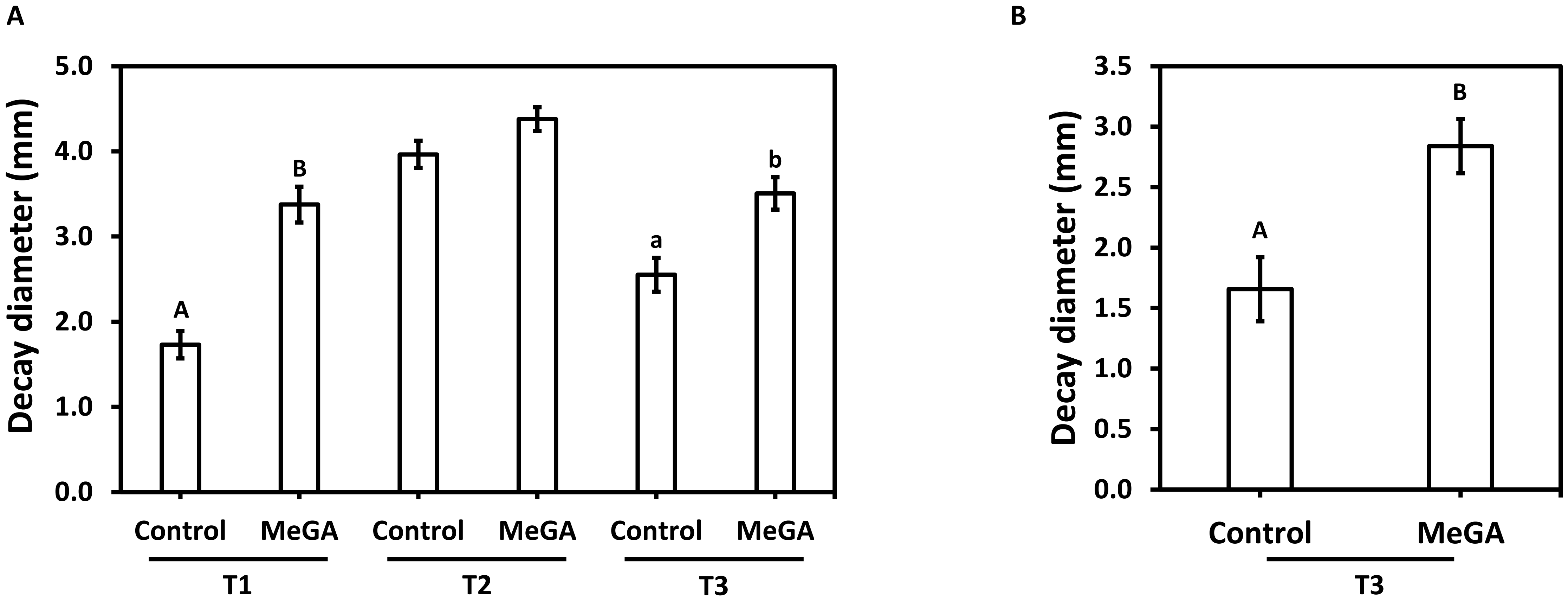

Figure S3. Effect of methyl gallic acid (MeGA) on the colonization of *A. alternata*. The fruits of Cvs. ‘Shinshu’ (A) and 'Triumph' (B) were inoculated with various combinations of MeGA and *Alternaria* conidia suspension. In T1, the fruits were pierced and inoculated with *A. alternata*, preincubated with 1 mM MeGA for 4 h. In T2, 1 mM MeGA was added to holes and followed by inoculation with *A. alternata*. In T3, the fruits were first dipped in 1 mM MeGA for 2 min, then pierced, and inoculated with *A. alternata.* Different letters indicate a significant difference between treatments according to Tukey-Kramer HSD and Student t-test $p \leq 0.05$.

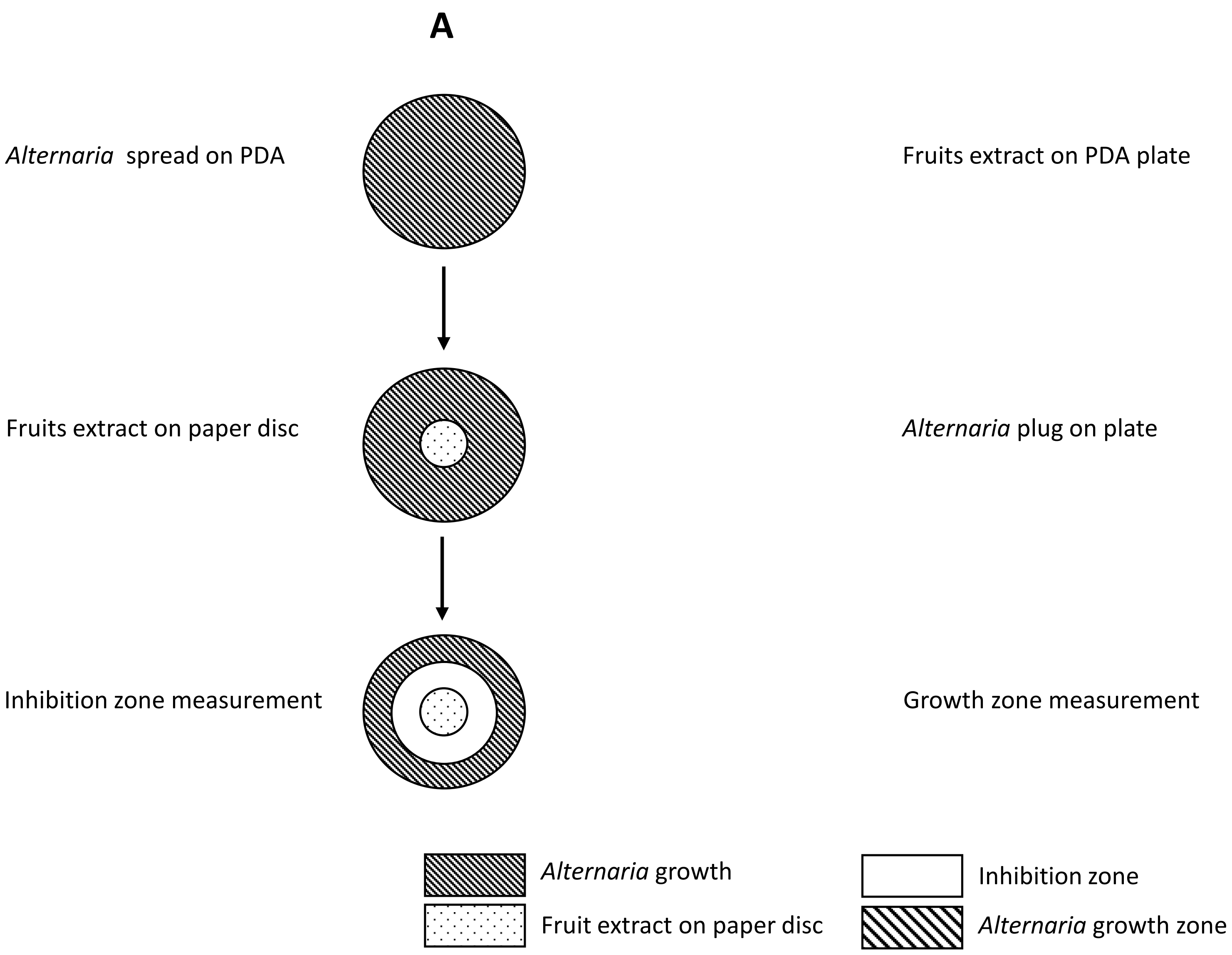

Figure S4. Schematic presentation of the methods used for the anti-*Alternaria* activity. Disc assay (A) and poisonous media assay (B).

Table S1. List of cultivars studied and harvest dates.

Cultivars type	Persimmon cultivars number	Persimmon cultivars name	Harvest date 2016	Harvest date 2017
Astringent (AST)	32	Jishi	20/09	02/10
	117	Qiyuezao	07/08	29/08
	121	Guilianqing shi	01/12	19/11
	181	Yuanxiao shi	01/12	19/11
		Triumph	04/11	05/11
	26	Lianhua shi	20/09	02/10
		Rojo Brillante	01/11	05/11
	27	Jixinhuang	20/09	16/10
	13	Jumi shi	01/11	02/10
	123	Raotianhong	14/08	29/08
Non-astringent (NAST)		Matsumoto Wasa Fuyu	01/11	05/11
		Late Fuyu Mutant	01/12	19/11
		Shinshu	20/09	02/10
		Ichikikei Jiro	20/09	16/10
		Yoho	20/09	16/10
		Maekawa Jiro	01/11	16/10

Table S2. Polyphenolic compounds and their peak area identified in the first platform. Metabolites of peel and pulp of 3 replications are displayed ±SE.

			Peel AST						Peel NAST					
**S.No.**	**Prefered names**	**Metabolites**	**Peel AST-1**	**Peel AST-2**	**Peel AST-3**	**Average**	**SD**	**SE**	**Peel NAST-1**	**Peel NAST-2**	**Peel NAST-3**	**Average**	**SD**	**SE**
1	Betulinic acid-like	Betulinic acid	371601	399679	383559	384946.33	14090.32	8144.69	63669	86842	67398	72636.33	12442.95	7192.46
2	Caffeoyl maslinic acid	Caffeoyl maslinic acid	357660	290468	404755	350961.00	57437.24	33200.72	98123	221086	145490	154899.67	62019.20	35849.25
3	Catechin	Catechin	1808025	2140525	1055823	1668124.33	555719.13	321224.93	1400740	871705	1236651	1169698.67	270797.84	156530.54
4	Chlorogenic acid_der?	Chlorogenic acid_der?	221039	198129	242503	220557	18118.81549	10473.30375	58622	66399	70927	65316	5081.53015	2937.300665
5	Chlorogenic acid_stn	Chlorogenic acid_stn	11966	8363	9548	9959	1499.353861	866.6785323	0	0	0	0	0	0
6	Coumaric acid	Coumaric acid	1284	2281	2151	1905.33	542.00	313.30	0	0	0	0.00	0.00	0.00
7	Dihydrophaseic acid-like	Dihydrophaseic acid	26719	27722	19733	24724.67	4351.90	2515.55	1769	1649	5610	3009.33	2253.04	1302.34
8	Epicatechin gallate	Epicatechin gallate	25331	25296	20771	23799.33	2622.67	1516.00	0	725	0	241.67	418.58	241.95
9	Ferulic acid -like	Ferulic acid	0	0	0	0.00	0.00	0.00	1618	0	6496	2704.67	3381.59	1954.68
10	feruloyl quinic acid-like	5-*o* -feruloyl quinic acid	32478	23432	27032	27647.33	4554.28	2632.53	21053	25653	54173	33626.33	17941.97	10371.08
11	Gallic acid	Gallic acid	137490	114989	79737	110738.67	29110.16	16826.68	0	0	2559	853.00	1477.44	854.01
12	Gallocatechin	Gallocatechin	226738	248251	200816	225268.33	23751.63	13729.26	151736	143815	197698	164416.33	29093.60	16817.11
13	Homogentisic acid	Homogentisic acid	0	20956	0	6985.33	12098.95	6993.61	0	0	0	0.00	0.00	0.00
14	Hyperin (quercetin 3 galactoside)	Hyperin (quercetin 3 galactoside)	1439559	4426569	303800	2056642.67	2129530.52	1230942.50	7441270	1650942	2176587	3756266.33	3202110.97	1850931.20
15	Kaempferol hexoside	Kaempferol glycoside	637270	2733890	87299	1152819.67	1396586.76	807275.58	1658624	350945	685072	898213.67	679395.41	392714.11
16	Luteolin-di-hexoside	Luteolin-3',7-di-*o* -glucoside	47749	92273	13996	51339.33	39261.81	22694.69	112375	34368	54572	67105.00	40485.55	23402.05
17	Luteolin-glucuronide	Luteolin-7-*o* -glucuronide	14112	35002	0	16371.33	17610.04	10179.21	42946	7520	13410	21292.00	18982.75	10972.69
18	Luteolin-malonylglucoside	Luteolin 7-*o* -(6"-malonylglucoside)	167520	99284	164012	143605.33	38423.46	22210.09	3343	5897	9424	6221.33	3053.45	1765.00
19	Methylgallic acid	4-*o* -Methylgallic acid	27300	18653	13205	19719.33	7107.75	4108.52	0	0	0	0.00	0.00	0.00
20	Monogalloyl-Hexoside	Monogalloyl-Hexoside	0	910	0	303.33	525.39	303.69	1135	2277	2644	2018.67	786.97	454.90
21	Myricetin rhamnoside	Myricitrin (*o* Rha_Myricetin)	1569063	4426569	303800	2099810.67	2112007.55	1220813.61	7441270	1650628	2178430	3756776.00	3201759.79	1850728.20
22	Naringenin-hexoside	Naringenin-7-*o* -glucoside	167974	156754	109547	144758.33	31005.65	17922.34	36539	25587	31213	31113.00	5476.68	3165.71
23	Phenylalanine	Phenylalanine	29425	12416	45503	29114.66667	13509.49351	7808.955786	0	759	0	253	357.7960313	206.8185152
24	Pomolic acid-like	Pomolic acid	398618	361954	480094	413555.33	60469.89	34953.69	322233	389577	348089	353299.67	33973.03	19637.59
25	Prephenate-like	Prephenate	146972	98891	80410	108757.67	34360.42	19861.51	0	0	0	0.00	0.00	0.00
26	Quercetin	Quercetin	6035	11130	2369	6511.33	4399.88	2543.28	8879	2490	4675	5348.00	3247.23	1877.01
27	Quercetin -galloylglucoside	Quercetin 3-(2-galloylglucoside)	221067	340898	100821	220928.67	120038.56	69386.45	99328	78548	108018	95298.00	15142.68	8753.00
28	Quinic acid-like	Quinic acid	442779	238237	554862	411959.33	160546.68	92801.55	105782	124127	119240	116383.00	9500.35	5491.53
29	Salicylic acid	Salicylic acid	4397	3798	4369	4188.00	338.04	195.40	952	691	1801	1148.00	580.38	335.48
30	Sinapyl alcohol hexoside	Syringin	10504	9634	6972	9036.67	1840.21	1063.70	5460	2388	3291	3713.00	1578.88	912.65
31	Vanillic acid	Vanillic acid	256212	175251	165411	198958.00	49826.92	28801.69	25418	18297	18487	20734.00	4057.58	2345.42
32	Vanilloyl-hexoside	1-*o* -Vanilloyl-beta-D-glucose	316465	259660	216070	264065.00	50342.25	29099.57	19953	11637	6499	12696.33	6789.27	3924.43

			Pulp AST						Pulp NAST					
**S.No.**	**Prefered names**	**Metabolites**	**Pulp AST-1**	**Pulp AST-2**	**Pulp AST-3**	**Average**	**SD**	**SE**	**Pulp NAST-1**	**Pulp NAST-2**	**Pulp NAST-3**	**Average**	**SD**	**SE**
1	Betulinic acid-like	Betulinic acid	871534	774816	823315	823221.67	48359.07	27953.22	50274	103553	166706	106844.33	58285.74	33691.18
2	Caffeoyl maslinic acid	Caffeoyl maslinic acid	0	0	0	0.00	0.00	0.00	0	0	0	0.00	0.00	0.00
3	Catechin	Catechin	91427	100776	106090	99431.00	7423.45	4291.01	361558	356236	497100	404964.67	79835.90	46147.92
4	Chlorogenic acid_der?	Chlorogenic acid_der?	24529	36474	30502	30501.66667	4876.525835	2818.801061	0	0	0	0	0	0
5	Chlorogenic acid_stn	Chlorogenic acid_stn	6290	6037	6984	6437	400.3406882	231.4108025	0	0	0	0	0	0
6	Coumaric acid	Coumaric acid	2129	2402	2909	2480.00	395.81	228.79	0	2514	0	838.00	1451.46	838.99
7	Dihydrophaseic acid-like	Dihydrophaseic acid	28815	26766	30369	28650.00	1807.16	1044.60	32284	14429	21470	22727.67	8993.70	5198.67
8	Epicatechin gallate	Epicatechin gallate	22134	19076	21457	20889.00	1606.18	928.43	0	0	0	0.00	0.00	0.00
9	Ferulic acid -like	Ferulic acid	0	0	0	0.00	0.00	0.00	0	4403	4501	2968.00	2570.83	1486.03
10	feruloyl quinic acid-like	5-*o* -feruloyl quinic acid	48765	33800	57130	46565.00	11819.57	6832.12	97553	79035	123945	100177.67	22569.75	13046.10
11	Gallic acid	Gallic acid	266921	230348	656848	384705.67	236390.53	136641.93	19130	6611	8966	11569.00	6653.05	3845.69
12	Gallocatechin	Gallocatechin	29284	52523	31448	37751.67	12838.03	7420.82	62753	72541	100755	78683.00	19731.48	11405.48
13	Homogentisic acid	Homogentisic acid	41370	42113	0	27827.67	24102.33	13931.98	0	0	0	0.00	0.00	0.00
14	Hyperin (quercetin 3 galactoside)	Hyperin (quercetin 3 galactoside)	23781	12633	12688	16367.33	6420.48	3711.26	0	10815	16718	9177.67	8478.42	4900.82
15	Kaempferol hexoside	Kaempferol glycoside	13773	8666	2292	8243.67	5752.14	3324.94	0	0	1004	334.67	579.66	335.06
16	Luteolin-di-hexoside	Luteolin-3',7-di-*o* -glucoside	0	0	0	0.00	0.00	0.00	0	0	0	0.00	0.00	0.00
17	Luteolin-glucuronide	Luteolin-7-*o* -glucuronide	0	0	0	0.00	0.00	0.00	0	0	0	0.00	0.00	0.00
18	Luteolin-malonylglucoside	Luteolin 7-*o* -(6"-malonylglucoside)	5147	9305	10643	8365.00	2866.04	1656.67	0	0	0	0.00	0.00	0.00
19	Methylgallic acid	4-*o* -Methylgallic acid	98243	100158	63997	87466.00	20347.29	11761.44	0	0	0	0.00	0.00	0.00
20	Monogalloyl-Hexoside	Monogalloyl-Hexoside	0	0	2444	814.67	1411.04	815.63	16029	2739	34342	17703.33	15867.89	9172.19
21	Myricetin rhamnoside	Myricitrin (*o* Rha_Myricetin)	23781	12633	12688	16367.33	6420.48	3711.26	0	10815	16773	9196.00	8502.90	4914.97
22	Naringenin-hexoside	Naringenin-7-*o* -glucoside	24016	21300	19545	21620.33	2252.65	1302.11	0	0	0	0.00	0.00	0.00
23	Phenylalanine	Phenylalanine	63260	40438	88431	64043	19600.88143	11329.98926	0	752	3628	1460	1563.445767	903.7258769
24	Pomolic acid-like	Pomolic acid	178	6558	58	2264.67	3718.62	2149.49	0	0	0	0.00	0.00	0.00
25	Prephenate-like	Prephenate	123157	108530	121232	117639.67	7947.70	4594.05	0	0	0	0.00	0.00	0.00
26	Quercetin	Quercetin	0	0	0	0.00	0.00	0.00	0	0	0	0.00	0.00	0.00
27	Quercetin -galloylglucoside	Quercetin 3-(2-galloylglucoside)	4569	0	1020	1863.00	2398.32	1386.31	0	0	0	0.00	0.00	0.00
28	Quinic acid-like	Quinic acid	165020	130510	182151	159227.00	26303.37	15204.26	163661	115134	114223	131006.00	28283.73	16348.98
29	Salicylic acid	Salicylic acid	3239	2116	2396	2583.67	584.55	337.89	854	784	3136	1591.33	1338.18	773.51
30	Sinapyl alcohol hexoside	Syringin	2631	216	2421	1756.00	1337.81	773.30	0	0	118	39.33	68.13	39.38
31	Vanillic acid	Vanillic acid	310257	333614	318308	320726.33	11864.81	6858.27	24802	18161	30592	24518.33	6220.35	3595.58
32	Vanilloyl-hexoside	1-*o* -Vanilloyl-beta-D-glucose	662368	731576	797385	730443.00	67515.63	39026.38	16617	15233	26588	19479.33	6195.06	3580.96

Table S3. Metabolites putatively identified in peel and pulp of persimmon fruit using UHPLC-TOF/MS analysis from the first platform.to expected masses.
Stn-represents identifications compared to authentic standards. Tentative identification represents putative annotation according

No	Name	Quantifier MZ	RT	Formula	Representative ion	Identification
1	Betulinic acid	455.3531	12.4	C30H48O3	M-H	tentative
2	Caffeoyl maslinic acid	633.3797	10.7	C39H54O7	M-H	tentative
3	Catechin	289.0718	3.8	C15H14O6	M-H	stn
4	Chlorogenic acid_derivative	399.0933	4.2	C16H18O9	(M+COOH)-	tentative
5	Chlorogenic acid_stn	353.0878	3.9	C16 H18 O9	M-H	stn
6	Coumaric acid	163.0401	4.9	C9H8O3	M-H	stn
7	Dihydrophaseic acid	281.1394	4.9	C15H22O5	M-H	tentative
8	Epicatechin gallate	441.0827	5.3	C22H18O10	M-H	stn
9	Ferulic acid	193.0506	5.7	C10H10O4	M-H	tentative
10	Feruloyl quinic acid	367.1035	0.77	C17H20O9	M-H	tentative
11	Gallic acid	169.0142	0.74	C7H6O5	M-H	stn
12	Gallocatechin	305.0667	1.35	C15H14O7	M-H	tentative
13	Homogentisic acid	167.035	5.1	C8H8O4	M-H	stn
14	Hyperin	463.0882	5.3	C21H20O12	M-H	stn
15	Kaempferol hexoside	447.0933	5.35	C21 H20 O11	M-H	tentative
16	Luteolin-di-hexoside	609.1461	5.2	C27 H30 O16	M-H	tentative
17	Luteolin-glucuronide	461.0725	5.4	C21 H18 O12	M-H	tentative
18	Luteolin malonylglucoside	579.0992	4.6	C24 H22 O14	(M+COOH)-	tentative
19	Methylgallic acid	183.0299	3.5	C8H8O5	M-H	tentative
20	Monogalloyl-hexoside	331.0749	0.65	C13H16O10	M-H	tentative
21	Myricetin rhamnoside	463.0882	5.3	C21H20O12	M-H	tentative
22	Naringenin-hexoside	433.114	5.2	C21H22O10	M-H	tentative
23	Phenylalanine	164.0717	0.99	C9H11NO2	M-H	stn
24	Pomolic acid	471.348	10.9	C30H48O4	M-H	tentative
25	Prephenate	271.0459	0.7	C10H10O6	(M+COOH)-	tentative
26	Quercetin	301.0354	6.2	C15H10O7	M-H	stn
27	Quercetin -galloylglucoside	615.0992	5.34	C28H24O16	M-H	tentative
28	Quinic acid	191.0561	0.7	C7H12O6	M-H	tentative
29	Salicylic acid	137.0244	5.7	C7H6O3	M-H	stn
30	Syringin	371.1348	5.5	C17H24O9	M-H	tentative
31	Vannillic acid_derivative	461.1301	4.85	C8H8O4	M-H	tentative
32	Vanilloyl-hexoside	329.0878	4.8	C14H18O9	M-H	tentative

Table S4. Polyphenolic compounds and their peak area identified in the second platform. Metabolites of peel and pulp of 2-3 replications are displayed ±SE.

		Peel AST						Peel NAST					
**S.No.**	**Metabolites**	**Peel AST-1**	**Peel AST-2**	**Peel AST-3**	**Average**	**SD**	**SE**	**Peel NAST-1**	**Peel NAST-2**	**Peel NAST-3**	**Average**	**SD**	**SE**
1	Benzyl alcohol-hexose-pentose	5.91	6.42	6.29	6.21	0.27	0.15	4.36	5.76	5.67	5.26	0.79	0.45
2	Ferulic acid-dihexose	6.16	0.82	1.57	2.85	2.89	1.67	4.83	6.32	6.32	5.83	0.86	0.50
3	Galloylglucose	6.18	10.06	10.60	8.94	2.41	1.39	6.07	6.18	6.06	6.10	0.07	0.04
4	Kaempferol	3.72	3.36	3.92	3.67	0.28	0.16	3.22	4.71	6.26	4.73	1.52	0.88
5	Kaempferol hexose	8.87	9.92	8.65	9.15	0.68	0.39	3.87	9.46	8.17	7.16	2.93	1.69
6	Kaempferol hexose I	9.01	9.43	8.16	8.87	0.65	0.37	3.64	9.87	8.63	7.38	3.30	1.91
7	Kaempferol-glucose-rhamnose	0.83	6.64	5.07	4.18	3.01	1.74	0.28	0.62	0.57	0.49	0.18	0.11
8	Kaempferol-hexose-deoxyhexose	6.44	5.98	5.50	5.97	0.47	0.27	2.54	7.21	5.97	5.24	2.42	1.40
9	Methyl-butanol-hexose-pentose	5.89	6.62	6.99	6.50	0.56	0.32	4.91	6.06	6.42	5.80	0.79	0.46
10	Naringenin-dihexose	6.43	8.39	8.52	7.78	1.17	0.68	5.45	6.62	6.45	6.17	0.63	0.36
11	Phloretin-di-C-hexose	5.81	7.72	8.05	7.19	1.21	0.70	4.13	6.10	5.92	5.38	1.09	0.63
12	Phloretin-trihexose	3.24	6.17	5.98	5.13	1.64	0.95	0.01	3.40	3.39	2.27	1.96	1.13
13	Procyanidin dimer	8.46	8.43	8.48	8.46	0.03	0.01	4.59	8.61	8.40	7.20	2.26	1.31
14	Procyanidin dimer I	6.50	6.33	6.22	6.35	0.14	0.08	3.59	6.71	6.41	5.57	1.72	1.00
15	Procyanidin trimer	6.51	6.57	4.57	5.88	1.14	0.66	1.95	6.82	6.25	5.01	2.66	1.54
16	Quercetin deoxyhexose-hexose-deoxyhexose	2.01	6.32	6.18	4.84	2.45	1.41	0.83	0.64	0.83	0.77	0.11	0.06
17	Quercetin hexose	10.03	9.94	9.09	9.69	0.52	0.30	5.08	10.96	9.91	8.65	3.14	1.81
18	Quercetin hexose I	9.87	10.41	9.50	9.93	0.46	0.27	5.05	10.53	9.67	8.42	2.95	1.70
19	Rutin	1.89	7.49	6.35	5.24	2.96	1.71	0.76	0.66	0.67	0.69	0.05	0.03

		Pulp AST					Pulp NAST					
**S.No.**	**Metabolites**	**Pulp AST-1**	**Pulp AST-2**	**Average**	**SD**	**SE**	**Pulp NAST**	**Pulp NAST-2**	**Pulp NAST-3**	**Average**	**SD**	**SE**
1	Benzyl alcohol-hexose-pentose	5.48	5.69	5.58	0.15	0.10	4.55	5.78	4.49	4.94	0.73	0.42
2	Ferulic acid-dihexose	0.23	0.54	0.38	0.21	0.15	4.36	1.26	3.32	2.98	1.57	0.91
3	Galloylglucose	10.49	10.46	10.47	0.02	0.02	5.94	10.18	6.22	7.45	2.37	1.37
4	Kaempferol	2.29	2.29	2.29	0.00	0.00	3.90	2.61	3.98	3.50	0.77	0.44
5	Kaempferol hexose	4.91	4.60	4.75	0.22	0.16	3.69	6.71	0.68	3.70	3.02	1.74
6	Kaempferol hexose I	4.18	3.45	3.81	0.52	0.37	2.89	6.40	3.83	4.37	1.82	1.05
7	Kaempferol-glucose-rhamnose	1.82	1.28	1.55	0.38	0.27	1.28	2.95	0.30	1.51	1.34	0.77
8	Kaempferol-hexose-deoxyhexose	0.83	2.01	1.42	0.84	0.59	0.35	4.52	2.54	2.47	2.09	1.21
9	Methyl-butanol-hexose-pentose	6.88	6.91	6.89	0.02	0.02	5.07	6.61	4.57	5.41	1.07	0.62
10	Naringenin-dihexose	7.58	7.59	7.59	0.00	0.00	5.41	8.10	5.55	6.35	1.51	0.87
11	Phloretin-di-C-hexose	6.57	6.44	6.51	0.09	0.06	3.51	7.78	3.62	4.97	2.43	1.41
12	Phloretin-trihexose	6.01	5.83	5.92	0.13	0.09	0.67	6.34	3.16	3.39	2.84	1.64
13	Procyanidin dimer	4.03	4.14	4.08	0.07	0.05	5.29	8.34	4.53	6.05	2.01	1.16
14	Procyanidin dimer I	2.17	2.61	2.39	0.31	0.22	3.72	6.15	2.51	4.13	1.85	1.07
15	Procyanidin trimer	1.77	0.79	1.28	0.69	0.49	3.02	6.36	2.25	3.88	2.19	1.26
16	Quercetin deoxyhexose-hexose-deoxyhexose	3.43	2.43	2.93	0.70	0.50	0.83	5.49	0.47	2.26	2.80	1.62
17	Quercetin hexose	4.97	3.81	4.39	0.82	0.58	4.08	7.90	3.77	5.25	2.30	1.33
18	Quercetin hexose I	5.70	4.82	5.26	0.63	0.44	4.41	8.30	4.47	5.73	2.23	1.29
19	Rutin	4.23	2.94	3.59	0.91	0.65	0.30	5.47	0.63	2.13	2.90	1.67

Table S5. Metabolites putatively identified in peel and pulp of persimmon fruit using UPLC/qTOF-MS from the second platform. Tentative identification represents putative annotation according to expected masses.

S. No.	Putative annotated metabolites	m/z detected [M-H]-	RT (min)	Fragments detected by MS^E	Molecular formula	Error (ppm)	UV (nm)
1	Benzyl alcohol-hexose-pentose	401.145	6.38	161.04	C18H26O10	3.2	na
2	Ferulic acid-dihexose	517.158	5.11	134.04	C22H30O14	0	na
3	Galloylglucose	331.064	1.22	271.04; 211.02; 169.01; 151.00	C13H16O10	5.9	277
4	kaempferol (S)	285.04	19.70	255.03	C15H10O6	1.8	na
5	Kaempferol hexose	447.092	12.05	285.04; 284.03; 255.03; 227.03	C21H20O11	0	264, 347
6	Kaempferol hexose I	447.092	11.39	285.04; 284.03; 255.03; 227.03	C21H20O11	5.4	264, 347
7	Kaempferol-glucose-rhamnose	593.151	11.61	285.039	C27H30O15	2	265, 359
8	Kaempferol-hexose-deoxyhexose	593.155	10.54	284.03	C27H30O15	2	na
9	Methyl-butanol-hexose-pentose	381.175	7.51	249.11	C16H30O10	0.8	na
10	Naringenin -dihexose	595.166	6.11	475.12; 415.09; 385.09; 355.08	C27H32O15	1.5	281
11	Phloretin-di-C-hexose	597.181	11.12	477.14, 459.13, 417.12, 387.11, 357.10, 345.05, 327.08, 315.08	C27H34O15	5	230, 286, 330sh
12	Phloretin-trihexose	759.231	8.25	651.19, 639.18, 621.18, 579.17, 549.15, 519.15	C33H44O20	5	285, 335 sh
13	Procyanidin dimer	577.134	4.08	451.10;425.08;407.07; 339.08;289.07;245.08; 161.02;125.02	C30H26O12	0.7	na
14	Procyanidin dimer I	577.134	8.04	451.10;425.08;407.07; 339.08;289.07;287.05;245.08; 161.02;125.02	C30H26O12	5.9	na
15	Procyanidin trimer	865.197	5.01	739.16;713.15;695.14;577.13;425.08;407.07	C45H38O18	3.5	na
16	Quercetin deoxyhexose-hexose-deoxyhexose	755.2	8.50	300.03	C33H40O20	1.6	256, 354
17	Quercetin hexose	463.086	10.08	301.03;300.02;178.99;151.01	C21H20O12	1.9	254, 352
18	Quercetin hexose I	463.087	10.41	301.03;300.02;178.99;151.00	C21H20O12	3.2	254, 352
19	Rutin (S)	609.145	10.01	301.03;300.03;271.02;255.03;179.00	C27H30O16	1.1	254, 352

(S) = commercial standard
